# Amyloid Beta Peptides Differentially Affect Hippocampal Theta Rhythms *In Vitro*

**DOI:** 10.1155/2013/328140

**Published:** 2013-06-25

**Authors:** Armando I. Gutiérrez-Lerma, Benito Ordaz, Fernando Peña-Ortega

**Affiliations:** ^1^Departamento de Neurobiología del Desarrollo y Neurofisiología, Instituto de Neurobiología, UNAM, Boulevard Juriquilla 3001, 16230 Querétaro, Mexico; ^2^Departamento de Farmacobiología, Cinvestav-IPN, Calzada de los Tenorios 235, Col. Granjas Coapa, 14330 México, DF, Mexico

## Abstract

Soluble amyloid beta peptide (A**β**) is responsible for the early cognitive dysfunction observed in Alzheimer's disease. Both cholinergically and glutamatergically induced hippocampal theta rhythms are related to learning and memory, spatial navigation, and spatial memory. However, these two types of theta rhythms are not identical; they are associated with different behaviors and can be differentially modulated by diverse experimental conditions. Therefore, in this study, we aimed to investigate whether or not application of soluble A**β** alters the two types of theta frequency oscillatory network activity generated in rat hippocampal slices by application of the cholinergic and glutamatergic agonists carbachol or DHPG, respectively. Due to previous evidence that oscillatory activity can be differentially affected by different A**β** peptides, we also compared A*β*_25−35_ and A*β*_1−42_ for their effects on theta rhythms *in vitro* at similar concentrations (0.5 to 1.0 **μ**M). We found that A*β*_25−35_ reduces, with less potency than A*β*_1−42_, carbachol-induced population theta oscillatory activity. In contrast, DHPG-induced oscillatory activity was not affected by a high concentration of A*β*_25−35_ but was reduced by A*β*_1−42_. Our results support the idea that different amyloid peptides might alter specific cellular mechanisms related to the generation of specific neuronal network activities, instead of exerting a generalized inhibitory effect on neuronal network function.

## 1. Introduction

Alzheimer's disease (AD) is a dementia of increasing prevalence [1], which is produced, at least in its early stages, by the extracellular accumulation of amyloid beta protein (A*β*) [2–4]. Early deterioration of hippocampal function, likely induced by soluble A*β*, contributes to the initial memory deficits observed in AD patients [4–8]. Interestingly, A*β* encompasses several peptide species which differ in their length, solubility, biological activity, toxicity, and aggregation propensity [3, 4, 9]. *Aβ*_1−40_ and *Aβ*_1−42_ are the most abundant A*β* peptides found in senile plaques and vascular deposits of AD patients [10, 11]; however, these deposits also contain A*β* peptides with shorter sequences such as *Aβ*_25−35_ [12–14]. *Aβ*_25−35_ can be produced in AD patients by enzymatic cleavage of *Aβ*_1−40_ at its hydrophobic C-terminus [14, 15], and it has been proposed that *Aβ*_25−35_ constitutes one of the biologically active fragments of A*β* [12, 16, 17]. Despite the extensive literature showing that the effects produced by *Aβ*_25−35_ are mostly reproduced by the full-length sequence [3, 12, 16, 18–28], other reports indicate that this is not always the case. For instance, it has been shown that the reduction in long term potentiation (LTP) produced by *Aβ*_1−40_ is not reproduced by *Aβ*_25−35_ [29]. In contrast, whereas *Aβ*_25−35_ induces intracellular actin aggregation and alters axonal transport, *Aβ*_1−42_ does not [30]. The same pattern occurs with the increase in intracellular calcium observed after application of *Aβ*_25−35_, which is not reproduced by *Aβ*_1−42_ [31]. It has already been proposed that different forms of soluble A*β* alter cognitively related, synchronized electrical oscillatory activity in neural circuits [9, 18, 19, 32–34]. Normal hippocampal function is strongly dependent on an oscillatory activity called theta rhythm [35–38] which, in lower mammals, includes oscillations ranging from 3 to 12 Hz [35–38]. Hippocampal theta rhythm participates in memory consolidation during REM sleep, in synaptic plasticity, in neural coding of place, and in spatial memory [8, 37, 39–43]. Interestingly, AD patients show alterations in theta rhythm activity [5, 44, 45]. Some reports indicate that cognitive dysfunction correlates with an increase of resting theta rhythm [5, 8, 44, 45], but other studies show a reduction of cognitive-induced theta rhythm [45]. Similar findings have been observed in transgenic mice that overproduce A*β* and exhibit AD-like symptoms [8, 46–50]. The complex relationship between AD pathology and theta rhythms can be explained by the theta rhythm heterogeneity that exists both in humans and in mice [51, 52]. Theta rhythms require the activation of either cholinergic or glutamatergic pathways, or both [53, 54], which are profoundly damaged in AD patients [55, 56] and severely compromised in transgenic AD mouse models [57–59]. Moreover, it is known that A*β* can affect both cholinergic [60, 61] and glutamatergic transmission [19, 62]. Thus, the application of A*β* in either the medial septum or in the hippocampus reduces theta rhythm both *in vivo* and *in vitro* [19, 33, 34, 63–65] and simultaneously induces cognitive deficits [33, 65]. Moreover, carbachol-induced theta rhythm generation is impaired in slices obtained from triple-transgenic AD mice [48]. Despite all this evidence, it is still unknown if A*β* can alter cholinergically and/or glutamatergically induced theta rhythms to the same extent *in vitro*. We have addressed this question and also evaluated the potential differences in biological activity between *Aβ*_25−35_ and *Aβ*_1−42_ on both cholinergically and glutamatergically induced theta rhythms, as was done earlier for beta rhythms [9]. We tested these peptides at concentrations of 1 *µ*M or less because most studies report A*β* concentrations in the low nM range both in AD patients [66–70] and in AD transgenic mice [71–74]. However, some reports show that A*β* in AD patients can reach high nM [75–77] or even *µ*M concentrations [66, 78, 79] (for a review see [80]). Our results show that *Aβ*_25−35_ reduces, with less potency than *Aβ*_1−42_, carbachol-induced field theta oscillatory activity. In contrast, DHPG-induced oscillatory activity was not affected by *Aβ*_25−35_ but was reduced by *Aβ*_1−42_.

## 2. Material and Methods

### 2.1. Animals

All experiments were performed using 7- to 10-week-old male Wistar rats. Animals used in this study were housed at 22°C and maintained on a 12-h:12-h light/dark cycle with free access to food and water. All the experimental protocols were approved by the Local Committees of Ethics on Animal Experimentation (CICUAL-Cinvestav and INB-UNAM). Experiments were performed according to the Mexican Official Norm for the Use and Care of Laboratory Animals (NOM-062-ZOO-1999).

### 2.2. Hippocampal Slice Preparation

Hippocampal slices were obtained as follows: the animals were anesthetized intraperitoneally with sodium pentobarbital (63 mg/Kg) and perfused transcardially with cold modified artificial cerebrospinal fluid (maCSF) of the following composition (in mM): 238 sucrose, 3 KCl, 2.5 MgCl_2_, 25 NaHCO_3_, and 30 D-glucose, pH 7.4, bubbled with carbogen (95% O_2_ and 5% CO_2_). After a maximum of 1 min of transcardial perfusion, the animals were decapitated, and the brains were removed and dissected in ice-cold artificial cerebrospinal fluid (aCSF) containing (in mM) 119 NaCl, 3 KCl, 1.5 CaCl_2_, 1 MgCl_2_, 25 NaHCO_3_, and 30 D-glucose, pH 7.4, continuously bubbled with carbogen. One cerebral hemisphere was glued to an agar block with a 30° inclination and mounted on a vibratome (The Vibratome Company, St. Louis, MO, USA). Horizontal slices (400 *µ*m thick) containing the hippocampal formation were cut and left to recover at room temperature for at least 90 min in aCSF continuously bubbled with carbogen.

### 2.3. Electrophysiological Recordings

The slices were transferred to a submerged recording chamber continuously perfused at 20 mL/min with oxygenated aCSF. The temperature was kept constant at 29 ± 2°C. Extracellular field recordings were obtained with suction electrodes filled with aCSF and positioned over hippocampal area CA1, *stratum pyramidale* [62, 81]. The signal was amplified and filtered (highpass, 0.5 Hz; lowpass, 1.5 KHz) with a wide-band AC amplifier (Grass Instruments, Quincy, MA, USA). The experimental protocol consisted of recording the spontaneous basal activity for 10 min before adding either the general cholinergic agonist carbachol (Cch) [20 *µ*M] or the metabotropic glutamate group I agonist (S)-3,5-dihydroxyphenylglycine (DHPG) [10 *µ*M] to the perfusion system in order to generate stable and persistent oscillatory activity [53]. Then, we added freshly dissolved *Aβ*_25−35_ to the perfusion system at two different concentrations: 0.5 *µ*M and 1 *µ*M [19]. In a different set of experiments, the freshly dissolved full peptide *Aβ*_1−42_ [0.5 *µ*M] was also tested [62]. We used the freshly dissolved inverse sequences *Aβ*_35−25_ [1 *µ*M] and *Aβ*_42−1_ [0.5 *µ*M] as negative controls. In some experiments, atropine [1 *µ*M], a broad-spectrum muscarinic acetylcholine antagonist, was added to the perfusion system to block carbachol-induced oscillations. The metabotropic glutamate receptor group 5 antagonist 2-methyl-6-(phenylethynyl) pyridine (MPEP) [25 *µ*M] was added to block the DHPG-induced oscillations. All drugs were dissolved in distilled water and were obtained from Sigma (Sigma-RBI, St. Louis, MO). The stock solutions were prepared at concentrations of at least 1000X. In most cases 1 *µ*L of distilled water, containing the drug, was applied to 1 mL of aCSF which, in our hands, does not affect its osmolarity.

### 2.4. Data Analysis

The recordings were stored on a personal computer with an acquisition system from National Instruments (Austin, TX, USA) by using custom-made software designed in the LabView environment. The recordings obtained were analyzed off-line using the program Clampfit (Molecular Devices). The full experiments were analyzed, and for quantification purposes, segments of 100 sec were analyzed every 10 min, using a Fast Fourier Transform Algorithm with a Hamming window. The power spectra of all segments were normalized to the basal spontaneous activity of each individual experiment or normalized to the control cholinergic or glutamatergic oscillatory activity. We also performed an Autocorrelation Function Estimate to test for the self cross-correlation (rhythmicity) of the signal on short (1 sec window) traces. All data are expressed as mean ± standard error of the mean (SEM). In most cases the data distribution was markedly skewed, and hence we used a Mann-Whitney Rank Sum Test with statistical significance denoted by *P* < 0.05, or a Kruskal-Wallis One-Way Analysis of Variance on Ranks followed by either Dunn's Method for Multiple Comparisons versus Control Group or for All Pairwise Multiple Comparison. Differences with statistical significance are denoted by *P* < 0.001.

## 3. Results

### 3.1. A*β* Peptides Inhibit Carbachol-Induced Hippocampal Theta Oscillatory Activity

**Figure 1:**
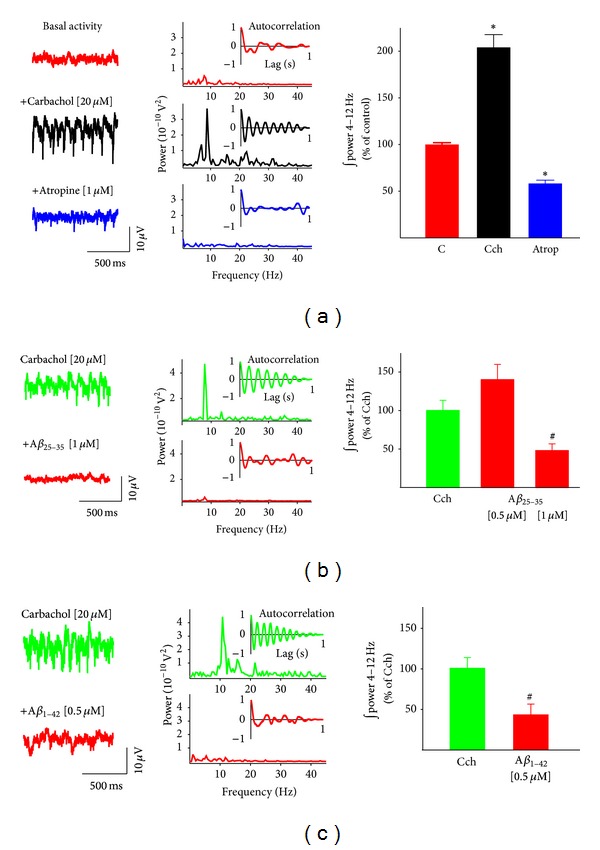
Amyloid beta peptides inhibit carbachol-induced hippocampal theta oscillatory activity. (a) Representative recordings (left) and the corresponding power spectra (right) of hippocampal population activity recorded in control conditions (upper trace and power spectrum) after bath application of carbachol (Cch 20 *µ*M; middle trace and power spectrum) and after the application of atropine 1 *µ*M (lower trace and power spectrum). The graph on the right shows the quantification of the integrated spectral power from 4 to 12 Hz. Note that the addition of Cch induces atropine-sensitive rhythmic oscillations that increase the power of the hippocampal population activity. (b) Representative recordings (left) and the corresponding power spectra (right) of hippocampal population activity recorded in the presence of carbachol 20 *µ*M (upper trace and power spectrum) and after bath application of *Aβ*_25−35_ 1 *µ*M (lower trace and power spectrum). The graph on the right shows the quantification of the integrated spectral power from 4 to 12 Hz before and after bath application of *Aβ*_25−35_ [0.5 *µ*M] and [1 *µ*M]. Note that a high concentration of *Aβ*_25−35_ inhibits Cch-induced rhythmic theta oscillations and that a lower concentration does not affect this activity. (c) Representative recordings (left) and their corresponding power spectra (right) of hippocampal population activity recorded in the presence of carbachol 20 *µ*M (upper trace and power spectrum) and after bath application of *Aβ*_1−42_  0.5 *µ*M (lower trace and power spectrum). The graph on the right shows the quantification of the integrated spectral power from 4 to 12 Hz before and after bath application of *Aβ*_1−42_. Note that *Aβ*_1−42_ inhibits Cch-induced rhythmic theta oscillations. The inset shown on each power spectrum is an autocorrelogram obtained from the corresponding trace. *indicates a significant difference with respect to control (*P* < 0.001), and ^#^indicates a significant difference with respect to the carbachol-induced oscillatory activity.

In order to study the effect of A*β* on cholinergically induced theta oscillatory activity, we first generated such activity by applying carbachol [20 *µ*M] to the perfusion system [82]. In these conditions, most of the slices (10 out of 13) generated field oscillatory activity with frequency components that fall into the theta range (Figure 1(a), middle trace) and have a peak frequency of 9.8 ± 0.39 Hz (Figure 1(a), middle power spectrum). The insets in the power spectra show the corresponding autocorrelation estimates of the hippocampal population activity, indicating that in the presence of carbachol [20 *µ*M] rhythmic population activity emerges. The rhythmicity exhibits a high degree of self-correlation (Figure 1(a)). Quantification of power in the classical theta range (4 to 12 Hz) [48, 53] shows that application of carbachol [20 *µ*M] increases the power of hippocampal population activity to 204.06 ± 13.79% of control basal activity (Figure 1(a), right graph). The activity described maintains stability for at least 100 min (*n* = 10). As expected, addition of atropine [1 *µ*M] completely abolished the oscillatory activity generated by carbachol and even reduced the power of the hippocampal activity below control levels, to 58.18 ± 3.64% of control basal activity (Figure 1(a)).

To test whether or not the amyloid peptides were capable of disrupting population theta oscillatory activity, we applied them to the bath after the application of carbachol. Addition of *Aβ*_25−35_ [0.5 *µ*M] to the perfusion system did not significantly change carbachol-induced theta oscillations (Figure 1(b)). However, it is important to notice that *Aβ*_25−35_ [0.5 *µ*M] shows a slight tendency to increase such activity (to 140.01 ± 19.86% of the cholinergic control (Figure 1(b); *n* = 8)). In the presence of *Aβ*_25−35_ [0.5 *µ*M] the peak oscillatory frequency remained unaffected (10.17 ± 0.78 Hz). Increasing *Aβ*_25−35_ concentration, in an independent group of slices, to 1 *µ*M reduced cholinergically induced theta oscillations to 47.93 ± 8.77% of the cholinergic control (Figure 1(b); *n* = 9). Likewise, the peak oscillatory frequency diminished significantly to 5.57 ± 1.26 Hz. Similarly, application of the full peptide *Aβ*_1−42_ [0.5 *µ*M] reduced the cholinergically induced theta oscillations to 42.60 ± 13.28% of the cholinergic control (Figure 1(c); *n* = 4); this inhibition in power was accompanied by a significant reduction in the peak oscillatory frequency to 6.45 ± 1.43 Hz.

**Figure 2:**
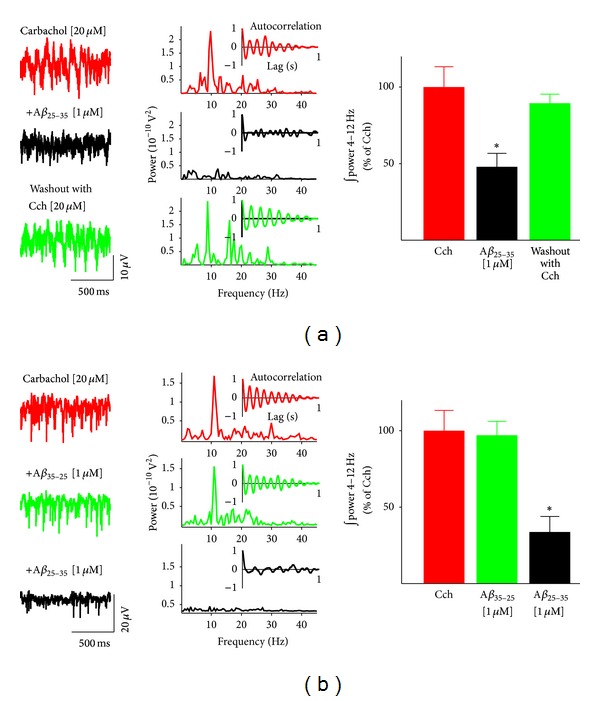
Amyloid beta inhibition of carbachol-induced hippocampal theta oscillatory activity is reversible and specific. (a) Representative recordings (left) and the corresponding power spectra (right) of hippocampal population activity recorded in the presence of carbachol 20 *µ*M (upper trace and power spectrum) after bath application of *Aβ*_25−35_ 1 *µ*M (middle trace and power spectrum) and after washout of A*β* (in the presence of carbachol 20 *µ*M; lower trace and power spectrum). The graph on the right shows the quantification of the integrated spectral power from 4 to 12 Hz. Note that the *Aβ*_25−35_-induced inhibition of theta activity is reversible upon washout. (b) Representative recordings (left) and the corresponding power spectra (right) of hippocampal population activity recorded in the presence of carbachol 20 *µ*M (upper trace and power spectrum) after bath application of the inverse sequence of *Aβ*_25−35_, *Aβ*_35−25_ [1 *µ*M] (middle trace and power spectrum) and after bath application of *Aβ*_25−35_ 1 *µ*M (in the presence of carbachol; lower trace and power spectrum). The inset shown on each power spectrum is an autocorrelogram obtained from the corresponding trace. The graph on the right shows the quantification of the integrated spectral power from 4 to 12 Hz. Note that the inverse sequence *Aβ*_35−25_ has no effect on the theta rhythm. *indicates a significant difference with respect to the carbachol-induced oscillatory activity (*P* < 0.001).

As an internal control, we washed out the *Aβ*_25−35_ [1 *µ*M] with aCSF containing carbachol [20 *µ*M] and found that the population theta oscillatory activity returned to nearly the same power level as before application of *Aβ*_25−35_ [1 *µ*M] (89.40 ± 5.95% of the cholinergic control; Figure 2(a), graph and lower trace; *n* = 4). The peak oscillatory frequency was also restored upon washout (8.56 ± 1.06 Hz; Figure 2(a), lower power spectrum; *n* = 4), and the rhythmicity of the theta oscillatory activity was identical to that observed before the application of *Aβ*_25−35_ [1 *µ*M] (Figure 2(a), upper inset). To test for the specificity of the effect produced by *Aβ*_25−35_, we used its inverse sequence *Aβ*_35−25_ [1 *µ*M] and found no effect on the carbachol-induced theta oscillations (Figure 2(b)). The power of the activity remained unaltered in the presence of *Aβ*_35−25_ (97.00 ± 9.16% of the cholinergic control, *n* = 10; Figure 2(b), graph and middle trace), and the peak theta frequency remained unaltered as well (8.49 ± 0.49 Hz, *n* = 10, Figure 2(b), middle power spectrum). Of course, subsequent application of *Aβ*_25−35_ [1 *µ*M] in the continued presence of *Aβ*_35−25_ (1 *µ*M) reduced the carbachol-induced theta oscillations to 33.71 ± 10.19% of the cholinergic control (Figure 2(b), lower trace and power spectrum). We also tested the effect of the inverse sequence *Aβ*_42−1_ [0.5 *µ*M] and found that this peptide sequence does not significantly alter carbachol-induced activity (81.57 ± 26.67% of the cholinergic control, *n* = 5).

### 3.2. A*β* Peptides Differentially Affect DHPG-Induced Delta/Theta Oscillatory Activity

**Figure 3:**
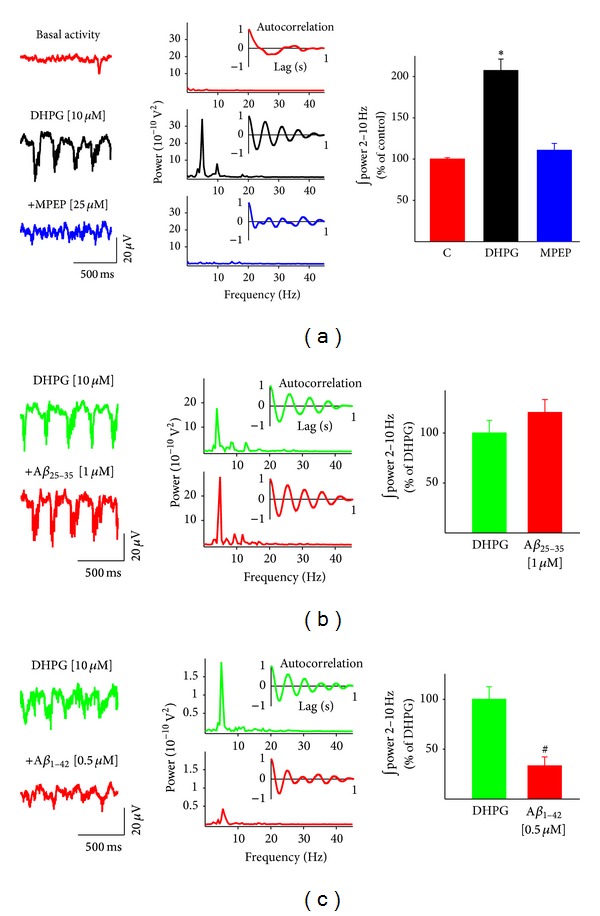
Amyloid beta peptides differentially inhibit DHPG-induced hippocampal theta oscillatory activity. (a) Representative recordings (left) and their corresponding power spectra (right) of hippocampal population activity recorded in control conditions (upper trace and power spectrum), after bath application of DHPG 10 *µ*M (middle trace and power spectrum) and after the application of MPEP 25 *µ*M (lower trace and power spectrum). The graph on the right shows the quantification of the integrated spectral power from 2 to 10 Hz. Note that DHPG induces MPEP-sensitive rhythmic oscillations that increase the power of the hippocampal population activity. (b) Representative recordings (left) and the corresponding power spectra (right) of hippocampal population activity recorded in the presence of DHPG 10 *µ*M (upper trace and power spectrum) and after bath application of *Aβ*_25−35_ 1 *µ*M (lower trace and power spectrum). The graph on the right shows the quantification of the integrated spectral power from 2 to 10 Hz. Note that *Aβ*_25−35_ does not affect DHPG-induced oscillatory activity. (c) Representative recordings (left) and the corresponding power spectra (right) of hippocampal population activity recorded in the presence of DHPG 10 *µ*M (upper trace and power spectrum) and after bath application of *Aβ*_1−42_  0.5 *µ*M (lower trace and power spectrum). The inset shown on each power spectrum is an autocorrelogram obtained from the corresponding trace. The graph on the right shows the quantification of the integrated spectral power from 2 to 10 Hz. Note that *Aβ*_1−42_ inhibits DHPG-induced rhythmic theta oscillations. *indicates a significant difference with respect to control (*P* < 0.001), and ^#^indicates a significant difference with respect to the DHPG-induced oscillatory activity.

As mentioned earlier, theta rhythm can also be induced by glutamatergic activation, mainly through metabotropic group I receptors [53, 83]. In order to study the effect of A*β* on glutamatergically induced theta oscillatory activity, we first generated such activity by applying DHPG [10 *µ*M] to the perfusion system. In these conditions, most of the slices (8 out of 10) generated oscillatory activity with frequency components slower than those present in cholinergically induced oscillatory activity, ranging from 2 to 10 Hz in the mixed delta/theta rhythm range (Figure 3(a); *n* = 8) with a mean peak frequency of 5.71 ± 0.92 Hz (Figure 3(a), middle power spectrum). The insets in the power spectra show the corresponding autocorrelations of the hippocampal population activity indicating that, in the presence of DHPG [10 *µ*M], rhythmic population activity emerges and is characterized by a high degree of self-correlation (Figure 3(a), inset). Quantification of power in the delta/theta range (2 to 10 Hz) shows that application of DHPG [10 *µ*M] increases the power of hippocampal population activity to 207.47 ± 13.66% of the control basal activity (Figure 3(a), right graph). This activity was stable for at least 100 min (*n* = 8). As expected, addition of MPEP [25 *µ*M] completely abolished the oscillatory activity generated by DHPG and reduced the power of the hippocampal activity to 110 ± 8.24% of control basal activity (Figure 3(a), lower trace and power spectrum). To test whether or not the amyloid peptides were capable of disrupting the glutamatergic population theta oscillatory activity, we added them after the application of DHPG. Addition of *Aβ*_25−35_ [1 *µ*M] to the perfusion system did not significantly change DHPG-induced delta/theta oscillations (120.52 ± 12.92% of the glutamatergic control; Figure 3(b), graph, lower trace and power spectrum). In the presence of *Aβ*_25−35_ [1 *µ*M], the peak oscillatory frequency remained unaffected (7.57 ± 0.64 Hz; *n* = 10). In contrast, application of full length *Aβ*_1−42_ [0.5 *µ*M] reduced DHPG-induced theta oscillations to 33.27 ± 8.92% of the glutamatergic control (Figure 3(c), graph, lower trace and power spectrum; *n* = 4). However, such inhibition in power was not accompanied by a significant reduction of the peak oscillatory frequency, which was 5.77 ± 1.12  Hz.

## 4. Discussion

The study of theta rhythm generation as well as its alterations in several neurological pathologies has received a great deal of attention [8, 84]. Such research has revealed that instead of a single theta rhythm, there actually exist a variety of rhythms that are involved in different behaviors and rely on different cellular mechanisms [52, 85]. In general, it has been proposed that theta rhythms are evoked through either cholinergic or glutamatergic means [8, 53, 84]. Our finding that A*β* affects both types of theta rhythms is of particular importance, given that both cholinergic and glutamatergic neurotransmission are necessary to generate cognitive-related theta network oscillatory activity (as reviewed by [84]), and therefore any pathological disruption in the normal function of either neurotransmitter system will generate cognitive dysfunction and, eventually, dementia. Previous research has shown that soluble A*β* affects the power, frequency, and structure of synchronized oscillatory activity, mainly theta and gamma rhythms, which are strongly related to cognition [9, 18, 19, 32–34, 64, 65] and that such activity is disrupted in AD patients [5, 44, 86, 87] and in AD transgenic mice [46–50]. As we have previously shown that theta generation *in vivo* is affected by both *Aβ*_25−35_ [19] and *Aβ*_1−42_ [34], we took this knowledge a step further and tested whether or not freshly dissolved A*β* affected the power and frequency of either cholinergically or glutamatergically induced hippocampal population theta oscillatory activity in slices *in vitro*. We found that different sequences of A*β* induce, with some differences in potency, a statistically significant reduction in power of cholinergic population theta oscillatory activity. In contrast, glutamatergically induced oscillatory activity was not sensitive to the application of *Aβ*_25−35_, whereas it was strongly inhibited by *Aβ*_1−42_. The mechanisms involved in the A*β*-induced reduction of both cholinergically and glutamatergically generated population theta oscillatory activity are not clear, and there are several cellular and molecular targets through which A*β* can pathologically interact to produce synaptic and cellular dysfunction (as reviewed in [3, 88]). However, very recent evidence indicates that A*β* affects the oscillatory activity of the hippocampus mainly in two ways: the first one is a generalized reduction of both glutamatergic and GABAergic transmission of presynaptic origin [19, 62, 89, 90], and the second involves alterations in specific subpopulations of GABAergic interneurons [33, 89–91]. Additionally, carbachol induces rhythmic oscillations of membrane potential in hippocampal neurons that allow them to engage in the theta oscillatory rhythm [92]. It has been shown that such subthreshold oscillations are abolished by A*β* when they are induced by carbachol induction [18] or through application of depolarizing DC current [19]. Regarding the biochemical mechanisms involved in this effect, both muscarinic acetylcholine M1/M3 receptors and group I metabotropic glutamate receptors modulate several cellular mechanisms by activating phospholipase C and protein kinase C (PKC) [83, 93]. It has been shown that the activation of PKC mediated by carbachol is disrupted by A*β* [94], whereas the activation of PKC induced by ACPD, a general metabotropic glutamate agonist, is completely unaffected by A*β* [94]. Another important factor to take into account is that, as it has already been suggested, carbachol and DHPG most probably generate oscillatory activity through the activation of different subsets of hippocampal interneurons [53], and, therefore, A*β* may pathologically alter the proper function of some, but not of other kinds of interneurons, which may also explain why DHPG-induced oscillatory activity is resistant to *Aβ*_25−35_. Another possible explanation for the higher sensitivity of carbachol-induced theta rhythm to bath application of A*β* compared to DHPG-induced theta rhythms has emerged from the recent finding that A*β* directly disrupts the M1 muscarinic receptor/G-protein interaction [95]. In contrast, to our knowledge, such direct disruption has not been demonstrated for metabotropic glutamatergic receptors. To finish with this point, it is important to mention that, just like the theta oscillatory activity generated with DHPG, potassium-induced hippocampal beta rhythm was reduced by *Aβ*_1−42_ but was resistant to *Aβ*_25−35_ [9]. In our previous studies, we had tested the changes in hippocampal population activity induced by *Aβ*_25−35_ at concentrations ranging from 300 nM to 3 *µ*M [9, 19], whereas *Aβ*_1−42_ has been tested in the range from 10 nM to 0.5 *µ*M [19, 62]. Based on our previous observations, and those from others, we decided to use a common concentration of both peptides of 0.5 *µ*M, which had already been shown to produce similar effects on hippocampal population activity [19].

In another series of experiments, we washed out the peptide *Aβ*_25−35_ [1 *µ*M] from the system with aCSF containing carbachol, and both peak frequency and power of the theta field oscillatory activity were completely restored; therefore, the reduction of field oscillatory activity induced by A*β* is completely reversible. We have shown previously that inhibition of hippocampal population activity induced by *Aβ*_25−35_ and *Aβ*_1−42_ is equally reversible [19, 62]. The reversibility of the effect of *Aβ*_25−35_ on theta rhythm observed here corroborates these previous findings and gives us confidence that the effects induced by *Aβ*_1−42_ are reversible as well [62]. The reversibility of the effects induced by A*β* suggests that soluble A*β* does not permanently damage the neural circuitry needed to generate theta oscillatory activity *in vitro*, and secondly, it strongly supports the idea that soluble A*β* produces synaptic impairment rather than irreversible synaptic loss *in vitro*. Finally, we also tested the A*β* inverse sequences as a control for the specificity of the biological activity of the A*β* peptides used; as expected, the inverse sequences had no effect at all on either the peak frequency or the power of the cholinergically induced population theta oscillatory activity. As additional proof that A*β* has biological effects that are sequence and conformation specific, in the presence of the inverse sequence *Aβ*_35−25_ we added into the perfusion system the regular sequence *Aβ*_25−35_ [1 *µ*M] which, unsurprisingly, significantly reduced the power of the cholinergic theta oscillatory activity to approximately one-third of control cholinergic values. Finally, in relation to the differences in biological activity between *Aβ*_25−35_ and *Aβ*_1−42_, the latter always produced a reduction of theta oscillatory activity, and, furthermore, it was more effective at a lower concentration than *Aβ*_25−35_, which is consistent with the idea that the most toxic A*β* species is soluble *Aβ*_1−42_ [96, 97], especially in its oligomerized form [98, 99]. In this study, both peptides were used in their soluble forms, which have been shown to be mainly composed of monomers and a few oligomers [100], although the precise concentrations of each aggregation species are hard to determine. Considering that the inhibition of beta rhythm hippocampal population oscillations produced by *Aβ*_25−35_ did not exhibit a clear concentration-dependent relationship in the low *µ*M range [9], we decided not to study higher concentrations. Given that *Aβ*_1−42_ was the most effective peptide used in terms of disrupting theta rhythm power, it would be of interest to test the effect of chemically oligomerized *Aβ*_1−42_ on population theta oscillatory activity induced both cholinergically and glutamatergically, since the oligomers should be even more potent at reducing the power of the theta rhythm at low concentrations.

## 5. Conclusions

In conclusion, A*β* peptides, but more potently *Aβ*_1−42_, can differentially reduce population theta oscillatory activity induced through either cholinergic or glutamatergic means, which suggests that instead of having a widespread effect, A*β* disrupts some particular mechanism of generation and maintenance of this activity. Importantly, this effect is sequence specific and completely reversible, suggesting that *Aβ* produces synaptic and cognitive impairments that could be potentially delayed or reversed.
